# Efficacy of intra-articular hypertonic dextrose prolotherapy versus normal saline for knee osteoarthritis: a protocol for a triple-blinded randomized controlled trial

**DOI:** 10.1186/s12906-018-2226-5

**Published:** 2018-05-15

**Authors:** Regina Wing Shan Sit, Ricky Wing Keung Wu, Kenneth Dean Reeves, David Rabago, Dicken Cheong Chun Chan, Benjamin Hon Kei Yip, Vincent Chi Ho Chung, Samuel Yeung Shan Wong

**Affiliations:** 1 Jockey Club School of Public Health and Primary Care, The Chinese University of Hong Kong, New Territories, Hong Kong; 2 Department of Physical Medicine and Rehabilitation (1986-2015), The University of Kansas, Kansas City, USA; 3 Department of Family Medicine, University of Wisconsin School of Medicine and Public Health, Madison, WI USA

**Keywords:** Dextrose prolotherapy, Normal saline, Randomized controlled trial, Knee osteoarthritis, Study protocol

## Abstract

**Background:**

Knee Osteoarthritis (KOA) is a very common condition with prevalence rising with age. It is a major contributor to global disability and has a large socioeconomic burden worldwide. Conservative therapies have marginal effectiveness, and surgery is reserved for severe symptomatic KOA. Dextrose Prolotherapy (DPT) is an evidence-based injection-based therapy for chronic musculoskeletal conditions including KOA. The standard “whole joint” injection method includes intra-articular injection and multiple extra-articular injections at soft tissue bony attachments. The procedure is painful and requires intensive procedural training often unavailable in conventional medical education, which potentially limits access. Intra-articular injection offers the possibility of a less painful, more accessible treatment. The aim of this project is to assess the clinical efficacy of intra-articular injection of DPT versus normal saline (NS) for KOA.

**Method:**

Seventy-six participants with KOA will be recruited from the community. We will conduct a single center, parallel group, superiority randomized controlled trial comparing DPT and NS injections, with blinding of physician, participants, outcome assessors and statisticians. Each group will receive injections at week 0, 4, 8 and 16. The primary outcome will be the Western Ontario McMaster University Osteoarthritis Index pain scale (WOMAC), and secondary outcomes include WOMAC composite score, the WOMAC function and stiffness subscale, the Visual Analogue Score of pain, objective physical function tests (the 30 s chair stand, 40- m fast paced walk test, the Timed up and go test) and the EuroQol-5D (EQ-5D). All outcomes will be evaluated at baseline, and 16, 26 and 52 weeks. All analyses will be conducted on an intention-to-treat basis using linear mixed regression models.

**Discussion:**

This paper presents the rationale, design, method and operational aspects of the trial. The findings will determine whether IA DPT, an inexpensive and simple injection, is a safe and effective non-surgical option for KOA. The results can be translated directly to clinical practice, with potentially substantial impact to patient care.

**Trial registration:**

The trial (ChiCTR-IPC-15006617) is registered under Chinese Clinical Trials Registry on 17^th^ June 2015.

## Background

Knee osteoarthritis (KOA) is a common chronic arthritis leading to joint pain and disability worldwide [1]. KOA is age-related [2]; by age 65, around 30% of the population has osteoarthritis [3, 4]. KOA is an expensive disease with significant socioeconomic burden due to its high prevalence, worker absenteeism and costly health care utilization [5, 6]. While exercise and weight reduction are effective in KOA, factors such as fatigue, accessibility and the arthritis itself have been identified as barriers for the actual participation [7]. Other conservative therapies such as physiotherapy, oral analgesic medications and complementary therapies such as acupuncture and herbal treatment have marginal effectiveness [8–11]. Total knee replacement (TKR) for advanced KOA is effective but costly [12].Thus, a safe and effective treatment option that complements the current conservative therapy remains a top priority in clinical practice and research [13, 14].

Pain and functional impairment in KOA are associated with a multifactorial set of degenerative intra-articular cartilage, bone and synovial knee structures, in addition to a complex interaction between genetic, biochemical, biomechanical, psychosocial, and other factors such as neurogenic inflammation and central pain sensitization [15–17]. The heterogeneous pain mechanisms in KOA may explain the variable responses to different therapies, and the search for effective non-surgical treatment for KOA has been challenging.

Dextrose prolotherapy (DPT) is an injection-based therapy for chronic musculoskeletal pain conditions including KOA [18]. The mechanism of action is likely multifactorial, and is hypothesized to work through stimulation of fibroblast and vascular proliferation, dense collagen deposition, and cartilage growth [19]. Additionally, dextrose solutions may have potential sensorineural analgesic effects as suggested recently by the effect of epidural injection of dextrose in the treatment of chronic non-surgical low back pain [20]. DPT may therefore treat KOA by targeting structural dysfunction, reducing nociceptive drive and minimizing peripheral sensitization.

The standard injection method of DPT involves a whole joint injection, consists of single intra-articular joint injection and multiple extra-articular injections at soft tissue attachments [21]. Pain and functional improvement in KOA have been reported in randomized controlled trials [22, 23], systematic review [24, 25] and meta-analysis [26, 27]. However, the procedure is painful due to multiple skin punctures, and premedication with a centrally acting opioid analgesic is sometimes used. In addition, the extra-articular injection protocol requires additional intensive postgraduate training, the access to which may be limited, minimizing availability of DPT.

The beneficial effects of a protocol using serial intra-articular dextrose injections have been reported in a small number of studies, though the results are limited by small sample size, lack of control arm or modest study design [28–32]. The current project aims to conduct a rigorous randomized controlled trial (RCT) to evaluate the intra-articular approach of DPT for KOA.

The primary aim of this study is to assess the clinical efficacy of intra-articular DPT versus normal saline (NS) in terms of self-reported knee pain at 52 weeks. We hypothesize that DPT is superior to NS in pain reduction as assessed by the Western Ontario McMaster University Osteoarthritis Index (WOMAC), a guideline recommended validated self-reported outcome in KOA trials [33]. Our secondary aims are to assess the clinical efficacy of intra-articular DPT versus NS in terms of subjective and objective functional improvement and quality of life at 52 weeks, using the WOMAC functional scale, 30 s chair stand, 40 m fast paced walk test, the Timed up and go test and EuroQuol-5D. EuroQuol-5D [34, 35].

## Methods

### Study Design

**Fig. 1:**
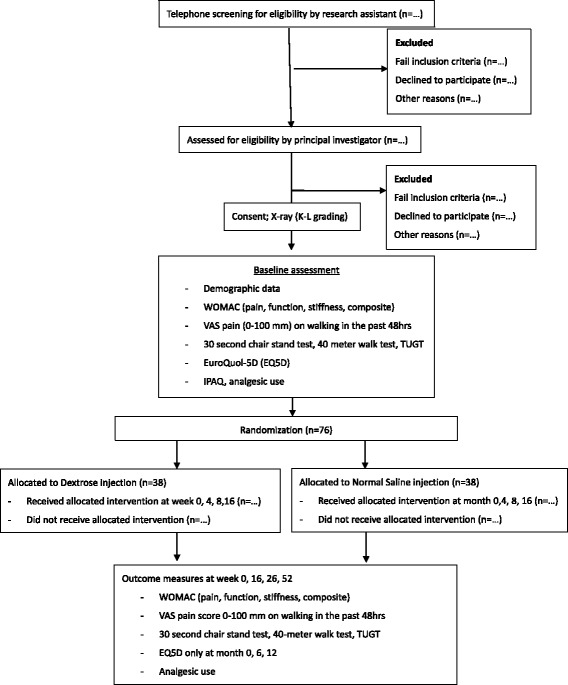
Study flow chart

The study is a 52-week, two-arm, parallel, superiority randomized controlled trial aimed to evaluate the comparative effectiveness of DPT versus NS for KOA. The study design follows the recommendation from the Osteoarthritis Research Society International (OARSI) [33], and the reporting will follow the Standard Protocol Items: Recommendations for Interventional Trials (SPIRIT) [36]. The study has been approved by the Joint Chinese University of Hong Kong – New Territories East Cluster Clinical Research Ethics Committee.(CREC no. 2014.059). This trial has been prospectively registered in the Chinese Clinical Trial Registry on 17^th^ June 2015. (Registration no. ChiCTR-IPC-15006617) The study workflow is summarized in Fig. 1.

### Eligibility

Eligibility will be screened by a trained research assistant using a phone interview, and potential eligible participants will be invited to meet the principal investigator (a physician) at the study site for confirming eligibility based on the following eligibility criteria:

Inclusion criteria include: (1) age ≥ 45 to ≤75 years old; we set the upper age limit to ensure adequate potential for cartilage regeneration [37]; (2) diagnosis of primary knee OA based on clinical and radiological criteria as defined by the American Rheumatology Association [38]; (3) moderate to severe knee pain for at least 3 months, defined as a score of ≥3 (0–6 ordinal response scale) on the question “What is the average level of your left/right knee pain in the past 3 months?”; and (4) failure to achieve pain reduction to a score < 3 (0–6 ordinal response scale) after 6 months of usual care, such as weight reduction, exercise, physical therapy and pharmacological treatment.

Exclusion Criteria include (1) history of corn allergy [39, 40]; (2) previous knee replacement surgery on the referring knee; (3) pregnancy; (4) body mass index (BMI) ≥ 35 kg/m^2^; (5) patients on anti-coagulant therapy; (6) prior knee injections within 3 months; (7) inflammatory or post-infectious knee arthritis, such as clinically diagnosed rheumatoid arthritis, gouty arthritis, psoriatic arthritis and septic arthritis; (8) significant effusion as defined by a ballotable patella; and (9) co-morbidity or lifestyle preventing participation in the study protocol.

### Recruitment and consent

Participants will be recruited in the General Outpatients Clinics (GOPCs) in the New Territories East (NTE) region of Hong Kong. There are seven general outpatient/family medicine clinics in NTE region that provide primary care services for the corresponding population, serving more than 1.2 million people. Subjects with KOA will be recruited via poster advertisement in GOPCs and direct physicians’ referral. The study site is a teaching clinic operated by the Chinese University of Hong Kong. After confirming the eligibility of the participants, the principal investigator will take approximately 15 min to describe the study goals, procedures, activities and possible alternatives, and answers all questions. Following this, interested prospective candidates will be given 7 days to consider the enrolment. The research assistant will then call the candidates for a second visit when written informed consent will be signed. After the enrolment, participants will receive a study identification number and undergo baseline data collection.

### Randomization and Allocation Concealment

Blocked randomization in 1:1 ratio will be used to allocate patients to the two groups [41]. The sequence will be generated by the Random Allocation Software. Random sequence will be concealed using sequentially numbered, opaque sealed envelopes (SNOSE) procedure [42]. These SNOSE will be kept by a person not involved in the care or evaluation of patients, or in the data analysis. The investigator enrolling the patient will have no access to SNOSE. The treatment allocation process starts when the investigator calls the personnel keeping the SNOSE. The computer database is designed in such a way that treatment allocation cannot be changed after randomization. Each participant will receive the SNOSE and they will be asked to sign on it. The SNOSE will only be opened at 52-week.

### Blinding of participants and personnel

Two registered nurses not involved in participant care will prepare the syringes with dextrose or NS identified only by study identification numbers. The syringes will be wrapped in aluminium foil to mask the solutions. The principal investigator and the study coordinator will therefore be blinded to group status of all subjects. The physician who conducts the injections will be blinded to the allocation group; he is also prohibited to communicate with the participants. Dextrose and saline solutions are odorless and identical in color and viscosity. Participants will be blinded to their group status, knowing only their randomization group number. To assess the success of participant blinding, participants will be asked to guess their group status at 12-month before unmasking, and data will be analyzed and interpreted using established procedure [43].

### Blinding in outcome assessment and data analysis process

All data collection will be performed by trained research assistants blinded to the allocation status of the patients via face to face interviews. They will receive rigorous training in standardized data collection procedures. Data entry personnel external to the research team will be employed to perform data entry such that the statistician can analyze data without the need to refer to allocation information, thus ensure blinding.

### Baseline measurement

Demographic data such as age, gender and BMI will be collected. Since objective functional outcome will be evaluated in this trial, the baseline physical activity status will be assessed using the Chinese version of International Physical Activity Questionnaire (IPAQ) [44]. Duration of knee pain and prior knee interventions such as weight reduction, knee exercise, physiotherapy, hyaluronic acid injection, corticosteroid injection or Traditional Chinese Medicine (TCM) etc. will be collected. All other co-morbidities will be documented as potential confounders. The severity of KOA will be graded by radiologist using the Kellgren-Lawrence Grading [45].

### Interventions

**Fig. 2:**
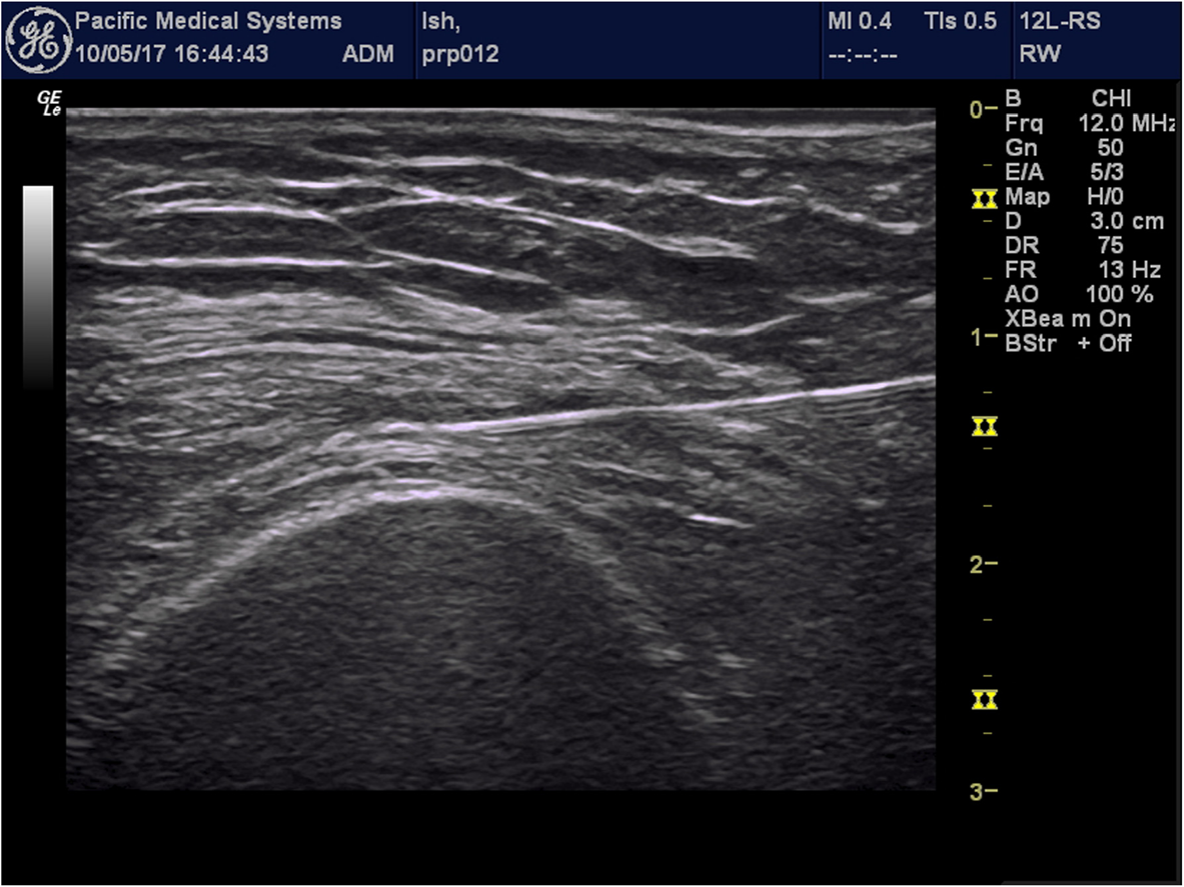
Suprapetella pouch injection under USG guidance

Following sterile preparation and injection of 1 ml 1% xylocaine as local anesthetic blebs, participants will be injected under ultrasound guidance with 25-gauge needle directed to the suprapatellar pouch using a linear probe with in plane approach. (Fig. 2) An ultrasound guided approach is used as it can guarantee injection into the joint space [46]. The injection procedures will be conducted under aseptic technique [47]. If both knees are painful, only the more painful knee will be injected. Injections will be conducted at week 0, 4, 8 and 16 for both groups. In case of pain flares after injection, the subsequent injection will be commenced after the flare is subsided, or at 1 month. If participants display allergic symptoms to the injected solution, therapy will be terminated but participants will continue to be followed in their allocated group until the end of the study.

Participants in the intervention group will receive injections with loaded syringe containing 5 ml 25% dextrose (D25), prepared by mixing 2.5 ml 50% dextrose with 2.5 ml sterile Water for Injection BP. D25 is a commonly used concentration for intra-articular DPT injection and has been used in previous studies [21, 22]. Participants in the control group will receive injections of 5 ml NS. Recent level one evidence has suggested that NS yields a statistically and clinically meaningful improvement in KOA-related pain up to 6 months after the injection. Therefore, NS will serve as the active control in this trial [48].

Post-injection care: Participants will be observed for 10 min post-intervention consistent with clinical practice and studies [21, 22]. Participants will be advised to take only acetaminophen (500 to 1000 mg every 4 to 6 h as-needed) and avoid non-steroidal anti-inflammatory drugs in the first 48 h after injection, which may interfere with the DPT mechanism of action. They will be allowed to take their usual analgesics medication after 48 h. Participants will be instructed on post-injection care and slow ramp-up of activity. Participants will be contacted 2 days after the injection to assess for side effects.

A home-based post-treatment quadriceps strengthening exercise will be prescribed to both groups and participants will be encouraged to practice daily. Co-interventions will be allowed to both groups, such as conventional medication, physical therapy, acupuncture, herbal medicine, over-the-counter drugs and other active treatments. The use of co-interventions will be retrieved from the Clinical Management System (CMS), an electronic system operated by the Hospital Authority in Hong Kong. Participants will be asked to recall their private treatment as well. However, participants are restricted to receive other injection therapies during the study period.

### Outcome measurement

#### Primary outcome

The Western Ontario McMaster University Osteoarthritis Index (WOMAC) pain score is the primary outcome. WOMAC is a disease-specific quality of life questionnaire for use in osteoarthritis clinical trials [33]. It consists of 24 self-reported items including knee pain (5 items), stiffness (2 items) and function (17 items). The subscales fulfill conventional criteria for content and construct validity, reliability, responsiveness and relative efficiency [49].

#### Secondary outcomes

Secondary outcomes include WOMAC composite score, the WOMAC function and stiffness score. Objective physical function is measured using the 30-s chair stand performance test, 40 m fast-paced walk test and the timed up –and-go test (TUGT). The three tests are recommended by the Osteoarthritis Research Society International (OARSI) as performance-based tests to assess physical function in people diagnosed with KOA [50]. Health related quality of life is assessed using the EuroQuol-5 questionnaire. The measure has strong construct validity, responsiveness and clinometric profile; it has been used to assess the economic impact of OA [35]. Joint pain will also be assessed by the Visual Analogue Score (VAS) in 0–100 mm scale upon walking in the past 48 h. Analgesic consumption will be assessed by the number of participants on analgesics by the 7-day recall diary. Before un-blinding, treatment satisfaction will be tested by asking “Would you recommend the therapy to others with knee OA like yours?”

### Data collection and management

Data will be collected at baseline, and at week 16, 26 and 52. We will record the number of potential candidates, responses received and their resolution, and the number of injection and assessment sessions attended. Follow up data will include the number at each follow up, the number of participants completing the trial, and the number of withdrawals due to all causes. Data entry, transfer and subsequent maintenance will be performed by a data manager. An electronic database will be used, and the server is in a physically secured location with backup on weekly basis. Access to study data is restricted to the study research team by username and password.

### Safety monitoring

Participants have a diary to document any discomfort after each injection; they are advised to call to the study coordinator if they are uncertain whether the discomfort is related to the injections. Standardization forms will be used for monitoring and reporting of side effects and adverse events. The principal investigator will be present in case of a significant adverse event. The principal investigator will report serious adverse events to the ethics committee within 24 h, and annual reports summarizing adverse events will be submitted to the Drug Office of the Department of Health, Hong Kong Administrative Region.

### Statistical issues

#### Sample size Calculation

The sample size calculation is based on 2 RCTs which evaluated the intra-articular injection of DPT and NS for KOA at 6 months [32, 51], assuming their therapeutic effect will remain stable for 1 year. The baseline characteristics of the two trials had comparable age, sex, BMI, and baseline WOMAC score. The mean (Standard Deviation) difference of WOMAC scores at 6 month compared with baseline status was 25.2 (20.3) points for DPT [32], and 9.53 (26.6) points for NS [51], respectively. Thus, assuming a pooled SD of 26.6, a sample size of 34 participants in each arm will have 80% power to detect a significant effect size of 0.70 in a two-sample t-test with alpha set at 0.05. Assuming 10% dropout rate, the total sample size is 76.

#### Data analysis

We will conduct linear mixed models to investigate significant changes over time for both primary and secondary outcomes following the intention to treat principle, i.e., all available data will be analyzed according to the group they are randomly assigned. The use of LMM also provides the means to include subjects with incomplete and use all available data to assess the treatment effect over time. In this model, intervention group, time, and the interactions between the intervention groups and time will serve as predictors with duration of knee pain and number of comorbidities as covariates. Given the longitudinal nature of the clinical trial data, we assume the autoregressive covariance structure will be the best fit for the data, but the statistical fitness by using other covariance structures will also be evaluated [52].

With a clearly defined target population, effectiveness and safety outcomes, and convenient data collection procedures, our trial should realize the goal of maximizing the number of participants who are maintained on the protocol-specified intervention until the outcome data are collected. In case of a large number of outcomes and covariates with missing data, we will use multivariate imputation using chained equations (MICE) to incorporate auxiliary information about the missing data. The imputation model will include prerequisite variables in the data analysis, variables for baseline socio-economic status, and also variables that are predictors of outcomes. About 10 iterations will be conducted in each imputation process with more iterations to be considered until the chain reaches convergence [53]. Twenty completed datasets will be imputed with the use of the chain equations. The Rubin’s rule will be applied to combine the effect estimates [54]. This approach provides estimated standard errors and *P* values that incorporate missing-data uncertainty. For the patients that meet any of the exclusion criteria such as alternative treatment or severe outcome, then all subsequent longitudinal measurements since that date of the event will be excluded from the analysis.

## Discussion

One of the objectives of International Association for the Study of Pain 2016 is to encourage research aimed at producing more effective and accessible treatment methods and outcomes for people with joint pain. Intra-articular injections remain a popular non-surgical treatment options for KOA. The current injection therapies include corticosteroids, hyaluronic acid and platelet rich plasma. Intra-articular corticosteroid is well known for its short-term pain relief up to 4 weeks only, and is usually indicated for acute inflammatory flares. Intra-articular hyaluronic acid appears to have longer pain relief up to 6 months, though its cost-effectiveness remains controversial. Intra-articular platelet rich plasma is emerging rapidly in recent years, but high quality scientific evidence of efficacy is lacking. In spite of the growing evidence of efficacy and effectiveness of DPT, it appears to be under-utilized in the medical world. Thus, there is a compelling need to conduct a high quality RCT with rigorous study design to determine the clinical efficacy of a brief intervention, IA DPT for KOA. IA DPT is safe, easily accessible and inexpensive; the injection can be easily performed by trained physicians. The trial follows the recommendations from the OARSI, and evaluates both validated self-reported and objective functional outcomes, as well as quality-of-life. Results will translate directly to clinical practice. Statistically and clinically positive outcomes on IA DPT would provide immediate, practical benefits to individual patients and society at-large through improved quality-of-life and decrease use of medical resources. Since dextrose is inexpensive, there is potential economic impact to the healthcare system in the management of KOA. Positive results from this high quality trial would suggest IA DPT could be used as non-surgical treatment for KOA.

## Abbreviations

ANCOVA: Analysis of covariance
ANOVA: Analysis of variance
BMI: Body mass index
CONSORT: CONsolidated standards of reporting trials
CREC: Clinical research ethics committee
D25: 5 ml 25% dextrose
DPT: Dextrose prolotherapy
EQ-5D: EuroQol-5D
GOPCs: General outpatients clinics
IPAQ: International physical activity questionnaire
KOA: Knee osteoarthritis
LMM: Linear mixed models
NS: Normal saline
NTE: New Territories East
OARSI: Osteoarthritis research society international
RCT: Randomized controlled trial
SNOSE: Sequentially numbered, opaque sealed envelopes
TCM: Traditional Chinese medicine
TKR: Total knee replacement
TUGT: Timed up –and-go test
VAS: Visual analogue score
WOMAC: Western Ontario McMaster University Osteoarthritis Index
